# Evidence-based recommendations for advancing the chronic pelvic pain syndrome management by the paradigm change from reactive symptom suppression to proactive 3PM-guided patient-centered care

**DOI:** 10.1007/s13167-026-00468-1

**Published:** 2026-08-28

**Authors:** Ousman Bajinka, Lamarana Jallow, Na Li, Christian Kuhn, Walther Kuhn, Ivana Stetkarova, Olga Golubnitschaja, Xianquan Zhan

**Affiliations:** 1 Shandong Provincial Key Laboratory of Precision Oncology, Shandong Cancer Hospital and Institute, Shandong First Medical University & Shandong Academy of Medical Sciences, 440 Jiyan Road, Jinan, Shandong 250117 P. R. China; 2 Department of Biochemistry, Cell and Molecular Biology, College of Basic and Applied Sciences, University of Ghana, Legon, Accra Ghana; 3 Department of Biology, Division of Physical and Natural Sciences, School of Arts and Science, University of The Gambia, Banjul, Islamic Republic of The Gambia; 4 Department of Gynaecology and Obstetrics, Würzburg University Hospital, Würzburg, Germany; 5 Department of Gynecology and Obstetrics, Donau-Isar Klinikum Hospital, University Medical Campus Lower Bavaria, Deggendorf, Germany; 6 Department of Neurology, Charles University, 3rd Faculty of Medicine and University Hospital FNKV, Prague 10, Srobarova 50, 10000 Czech Republic; 7 Predictive, Preventive and Personalised (3P) Medicine, University Hospital Bonn, Rheinische Friedrich-Wilhelms-University of Bonn, Venusberg Campus 1, Bonn, 53127 Germany; 8 Shandong Provincial Key Medical and Health Laboratory of Ovarian Cancer Multiomics, & Jinan Key Laboratory of Cancer Multiomics, School of Pharmaceutical Sciences, Shandong First Medical University & Shandong Academy of Medical Sciences, 6699 Qingdao Road, Jinan, Shandong 250117 P. R. China

**Keywords:** Chronic pelvic pain syndrome, Chronic sterile inflammation, Pain chronification, Interstitial cystitis & bladder pain syndrome, Chronic abacterial prostatitis, Vaginal dryness, Therapy-resistant prostate cancer, Predictive preventive personalized medicine (PPPM / 3PM), Multi-factorial disease, Poor outcomes by reactive care, Systemic mitochondrial stress, Homeostasis and biosensorics, Tear fluid-based diagnostics, Sympathetic overdrive syndrome (SOP), Holistic approach, Patient phenotyping and stratification, Digital health monitoring, Nutraceuticals, Microbiome, TRP channels, Treatments tailored to individualized patient profiles

## Abstract

Chronic pelvic pain syndrome (CPPS) encompasses a heterogeneous group of debilitating conditions including interstitial cystitis/bladder pain syndrome (IC/BPS), chronic abacterial prostatitis, endometriosis, and pelvic congestion syndrome, among others. Despite advances in symptomatic management, CPPS remains challenging due to its multifactorial etiology, complex phenotypes, and poor response to conventional reactive treatments. On the other hand, sympathetic overdrive phenotype (SOP) carriers frequently suffer from increased stress sensitivity, chronic sterile inflammation, pain chronification, and mitochondrial stress – all considered the key CPPS/SOP shared pathomechanisms. Further, the dominant vasoconstriction, altered sense regulation (e.g. the reduced feeling of thirst potentially resulting in systemic dehydration) as well as altered multi-drug resistance protein profiles characteristic for SOP (e.g. exemplified by the Flammer syndrome) may predispose affected individuals to the therapy resistance such as CPPS patients with vulvar-vaginal dryness and abacterial prostatitis. This review article highlights the central role of SOP, systemic mitochondrial stress as well as gut and urinary microbiome alterations – all, per evidence, are considered systemic modifiable risk factors of the CPPS manifestation, disease progression, and therapy resistance. The article introduces 3PM-guided patient-centered solutions aiming to improve life quality and individual outcomes in the CPPS patient cohort. Non-invasive mitochondria-based biosensorics, e.g. applied via the tear fluid analysis, and digital health monitoring, per evidence, enable early health risk assessment and patient stratification at the level of reversible damage to health. Recommended targeted preventive strategies encompass individually adapted lifestyle modifications, modulation of the stress-associated mitochondrial homeostasis, and application of supportive nutraceuticals. Personalized 3PM-guided treatment algorithms transform CPPS management into the patient-centered, cause-oriented and cost-effective proactive care, protecting vulnerable individuals against health-to-disease transition and preventing disease progression in stratified patients.

## Preamble

Chronic pelvic pain syndrome (CPPS) is a complex, multifactorial condition that affects millions of individuals worldwide, with a disproportionate burden towards women vs. men across all age groups [1, 2]. CPPS is not a single disease but a syndrome encompassing several distinct yet overlapping disorders, including interstitial cystitis/bladder pain syndrome (IC/BPS), chronic prostatitis/chronic pelvic pain syndrome (CP/CPPS), endometriosis, adenomyosis, pelvic congestion syndrome, vulvodynia, and pelvic floor tension myalgia [3, 4]. These conditions share common features: persistent non-cyclic pain localized to the pelvis for at least three to six months, significant negative impact on quality of life, frequent comorbid functional syndromes (irritable bowel syndrome, fibromyalgia, and chronic fatigue syndrome), and a tendency to relapse and flare [5, 6].

The pathophysiology of CPPS is notoriously heterogeneous. Traditional models have focused on peripheral organ pathology, such as bladder inflammation in IC/BPS or ectopic endometrial implants in endometriosis. However, these mechanisms fail to fully explain the widespread nature of symptoms, the lack of correlation between pathology and pain intensity, and the frequent overlap among different CPPS diagnoses as well as therapy resistance [7, 8]. Further, CPPS is recognized as a disorder involving central sensitization and autonomic nervous system imbalance [9]. Contextually, the topic-dedicated expert group of the European Association for Predictive, Preventive, and Personalized Medicine (EPMA) recently published a research article considering CPPS in the context of the Sympathetic Overdrive Phenotype (SOP) and concluding that the clinical manifestation of CPPS in the SOP carriers, to a large extent, is linked to the systemic modifiable phenotype-associated risk factors. An important message by the research group is: being characteristic for the phenotype, significantly increased stress sensitivity (e.g. psychologic, hormonal, temperature, environmental, and metabolic stress, among others) provokes mitochondrial stress followed by the chronification of sterile inflammation and pain. To this end, for the CPPS patients suffering specifically from the abacterial prostatitis (AP) and characterized by pain, voiding and sexual dysfunction persisting for over 3 months, the cold stress provocation appears to be one of the most specific disease triggers resulting in the CPPS manifestation [10]. Further, high sensitivity to the cold stress provocation, a diminished thermoregulation and predisposition to a spectrum of severe pathologies, are abundantly discussed in the scientific literature for the Flammer syndrome phenotype (SOP) carries [11]. Finally, in combination with an altered multi-drug resistance protein expressions demonstrated for the Flammer syndrome (SOP carriers) [12], these systemically dysregulated pathways are highly probable to cause resistance to conventional therapy requiring, therefore, a completely new strategy for more effective phenotype-specific prevention measures and disease management [13, 14]. Those epigenetically created systemic risks represent a promising target for holistic approaches with a high potential to change the paradigm from less effective reactive healthcare model to the advanced 3PM-guided proactive medical care [13]. This article aims to provide robust scientific evidence for the recommended paradigm shift from the currently applied reactive symptom suppression to proactive care utilizing conceptual and technological innovation comprising predictive diagnostics, individualized protection against health-to-disease transition and treatments tailored to individualized patient profiles. Corresponding roadmap for the clinical implementation is presented for consideration by relevant multi-professional groups.

### Literature search strategy

To ensure a comprehensive, transparent, and reproducible synthesis of evidence on the predictive, preventive, and personalized medicine (3PM) approach to chronic pelvic pain syndrome (CPPS), we conducted a systematic literature search informed by the principles of the Preferred Reporting Items for Systematic Reviews and Meta-Analyses (PRISMA) framework, adapted for the scope of a narrative review. The search strategy was designed to capture peer-reviewed articles addressing the pathophysiology, predictive diagnostics, preventive interventions, personalized treatments, and emerging technologies (e.g., multi-omics, digital biomarkers, and artificial intelligence) relevant to CPPS and its major subtypes.

Two reviewers (O.B. and L.J.) independently screened titles and abstracts, then full texts, against predefined eligibility criteria (original research, reviews, and clinical studies in English, published between 2000 and 2025, focusing on CPPS pathophysiology, biomarkers, prevention, or treatment). Disagreements were resolved by consensus or by a third reviewer (N.L.). The reference lists of included review articles were hand-searched for additional relevant studies.

A total of 1,247 unique records were identified initially. After removal of duplicates (*n* = 389), 858 records were screened. Of these, 712 were excluded at title/abstract level (mainly off-topic or non-original research). The remaining 146 full-text articles were assessed for eligibility, and 113 studies met the final inclusion criteria. These form the evidence base for the references cited in this review, covering key mechanistic, diagnostic, preventive, and therapeutic aspects of CPPS within the 3PM framework.

## Systemic effects and holistic approach to the CPPS modeling in 3PM framework

### Sympathetic overdrive phenotype (SOP) carriers are strongly predisposed to CPPS manifestation

Chronic pelvic pain syndrome (CPPS) represents a complex, multifactorial condition whose pathogenesis remains incompletely understood. Emerging evidence increasingly points to the autonomic nervous system; particularly sympathetic hyperactivity, as a critical driver in both the initiation and chronification of pelvic pain. The sympathetic overdrive phenotype (SOP), defined as the sustained dominance of sympathetic over parasympathetic tone, differs fundamentally from brief adaptive fight-or-flight” responses [13]. This chronic sympathoexcitation creates a systemic milieu that profoundly predisposes affected individuals to CPPS. Suprasegmental and segmental autonomic nervous system disorders partly explain pain chronification mechanisms [15].

Currently, the best-described SOP-relevant subpopulation comprises Flammer Syndrome phenotype (FSP) carriers, typically characterised by chronication of transient sympathoexcitation [13]. FSP carriers are success-oriented and highly stress-sensitive individuals; among medical students, FSP carriers may reach up to 70% [13]. While this attitude enables successful careers, it simultaneously exposes individuals to significant health risks. Key mechanisms linking SOP to CPPS include dominant vasoconstriction, altered sense regulation (e.g. reduces feeling of thirst potentially resulting in dry mouth syndrome and systemic dehydration), stress and pain chronification, chronic inflammation, matrix metalloproteinases hyperactivation, impaired wound healing, mitochondrial stress, and insufficient energy supply for repair [13]. Moreover, altered multi-drug resistance protein profiles characteristic for the FSP carriers may predispose affected individuals to the therapy resistance, e.g. in patients with vaginal dryness, abacterial prostatitis and prostate cancer [16–18].

Persistent vasoconstrictor drive perturbs cellular metabolism and reshapes tissue interfaces. Neuro-inflammation and sensitization of sensory nerves lead to persistent inflammation and pain, while central sensitization lowers pain thresholds. FSP carriers exhibit extreme stress sensitivity, perfectionism, prolonged sleep onset, reduced feeling of thirst, and increased pain sensitivity. The diminished feeling of thirst may result in systemic dehydration, further compromising pelvic tissue homeostasis [12]. Chronically increased endothelin-1 and mild hyperhomocysteinaemia synergistically cause progressing small vessel disease, systemic inflammation and mitochondrial stress chronification [13].

The recognition that SOP carriers are predisposed to CPPS opens new avenues for predictive, preventive, and personalized medicine (PPPM/3PM). Since key pathways involved in health-to-disease transition are epigenetically regulated and modifiable, they represent promising targets for holistic proactive approaches [13]. Patient stratification through advanced phenotyping; particularly FSP screening, plays a crucial role in predicting pain-associated outcomes and enabling targeted prevention of pain chronification [15].

### Mitochondrial stress and altered homeostasis in SOP carriers: the evident relevance for CPPS manifestation

Mitochondria do function as biosensors being reactive to stimuli and protective against health-to-disease transition [19]. They play key roles in signaling pathways decisive for cell fate, stress response, redox balance, immunity, and severity of disorders [20, 21]. Yet, no any health or disease condition was reported, which mitochondrial functionality would be irrelevant to [20]. In the context of SOP and CPPS, mitochondrial stress and impairments emerge as the central regulatory and patho/physiological mechanisms [13]. The relationship between SOP and mitochondrial stress is bidirectional and self-perpetuating. Chronification of parasympathetic-sympathetic imbalance leads to chronic ischemic events and progressing tissue damage associated with cyclic ischemia-reperfusion. This creates unstable oxygen supply leading to increased oxidative stress in mitochondria, reducing energy production. Chronically increased endothelin-1 accompanied, for example, with a mild hyperhomocysteinaemia synergistically are able to cause small vessel disease, systemic inflammation and mitochondrial stress chronification, potentially resulting in mitochondrial burnout [13]. Systemic mitochondrial stress extension is objectively measurable utilizing qPCR targeted to the cell-free mtDNA, which is instrumental for the 3PM-guied approach [21]. Indeed, mitochondrial stress relevance for CPPS manifestation and progression is increasingly recognized in prostatitis, and mitochondrial kinases have been identified as potential key proteins in the CPPS-prostatitis treatment [22]. Melatonin effectively relieves CPPS-related symptoms through mitochondrial pathways [22]. Cell plasticity modulation; sharing mechanistic parallels with chronic pain pathology, involves NF-κB signaling regulated by the mitochondrial redox status. Flavonoid modulation of NF-κB negatively affects cellular processes contributing to acquired plasticity [18]. This has implications for CPPS management, as targeting mitochondrial homeostasis and plasticity may mitigate the inflammatory milieu perpetuating pelvic pain. Under chronic sympathetic overdrive, mitochondrial adaptive capacity becomes overwhelmed, contributing to pain chronification and transition from acute protective signaling to persistent debilitative conditions. Nutraceuticals demonstrate health-promoting and illness-preventing properties with great therapeutic potential and safety profiles. However, application is beneficial only if meeting individual needs; health risk assessment and individualized patient profiles are pivotal, followed by application of adapted nutraceutical sets [20]. A patient-friendly non-invasive approach for monitoring mitochondrial homeostasis is established, utilizing tear fluid multi-omics, mitochondria as biosensors, and AI-based data interpretation [19, 21]. Further, tear fluid examination of mtDNA offers advantages over blood analysis due to more stable molecular patterns reflecting systemic effects as well as non-invasive collection [21]. Corresponding check-up tests is a powerful tool for holistic proactive medical services, health status and therapy efficacy monitoring [21].

In conclusion, systemic mitochondrial stress and altered mitochondrial homeostasis in SOP carriers represent evident modifiable risks for the CPPS manifestation and progression. Therefore, a holistic 3PM-guided approach, which encompasses comprehensive AI-based phenotyping, non-invasive mitochondrial health monitoring, and treatments tailored to individualized patient profiles, is strongly recommended for the paradigm shift from reactive to proactive medical services [13, 15, 18–21].

### Central sensitization and widespread pain

A significant portion of CPPS patients exhibit widespread pain beyond the pelvic region. Validation studies using simple body maps (13 regions) have shown that the presence of widespread pain identifies a distinct phenotype with worse symptom severity, poorer psychosocial functioning, and different treatment responses compared to patients with pain localized to the pelvis [23]. Peak alpha frequency (PAF) on electroencephalography is lower in patients with widespread pain, suggesting central sensitization as a neural biomarker [24]. Resting-state functional connectivity within the left frontoparietal network can predict short-term (3-month) pain reduction with 73% accuracy, offering a neuroimaging-based tool for personalized prognosis [25].

**Fig. 1:**
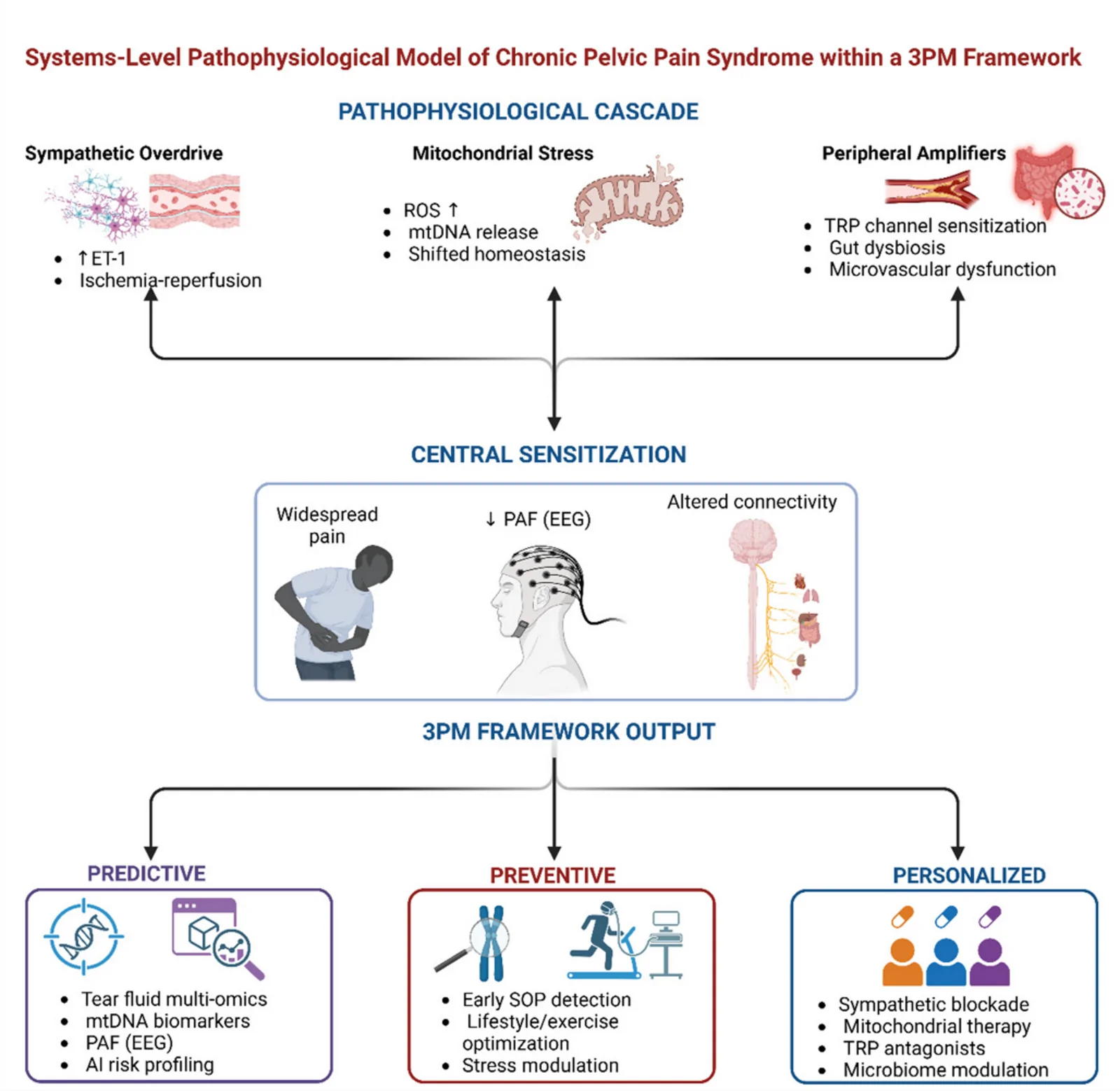
Systemic effects in the CPPS manifestation and 3PM-guided conceptual and technological innovation for the paradigm change from reactive to proactive medical services; Sympathetic overdrive, mitochondrial stress, and peripheral amplifiers (TRP channel sensitization, gut dysbiosis, and microvascular dysfunction) constitute key upstream mechanisms. These processes converge to drive central sensitization, characterized by widespread pain, reduced peak alpha frequency (PAF), and altered neural connectivity. The 3PM-guided approach consolidates essential pillars, namely advanced population screening and predictive diagnostics (comprehensive AI-based health risk assessment), targeted prevention (mitigation of multi-factorial risks), and treatments tailored to individualized patient profiles (stabilization of sympathetic-parasympathetic balance, modulation of mitochondrial homeostasis and microbiome profiles, application of TRP antagonists, amongst others)

Figure 1 summarizes systemic effects and innovative 3PM-guided holistic approach proposed for the CPPS management; the approach utilizes 3PM-relevant conceptual and technological innovation.

## Predictive diagnostics: from reactive to proactive care

### Clinical and biopsychosocial risk stratification

Early identification of individuals at risk for CPPS is the cornerstone of 3PM. A simple clinical test; cervical motion tenderness (parametropathy), has been validated as a diagnostic tool for CPPS in women, with 96.7% sensitivity and 92.8% specificity [26]. This test, performed during routine bimanual examination, can transform CPPS from a diagnosis of exclusion to a positive clinical finding. Similarly, impaired ability to relax pelvic floor muscles after voluntary contraction, particularly in men with ejaculation-related pain, distinguishes CP/CPPS patients from healthy controls [27].

Childhood trauma, especially violent trauma, is strongly associated with heightened pain sensitivity in adult CPPS patients, mediated by generalized sensory sensitivity [28]. Early and recent life adversity also correlate with elevated LPS-stimulated cytokines (IL-6, TNFα, IL-1β) in women with IC/BPS, indicating that adverse life events should be assessed as risk factors for inflammation [29]. Incorporating trauma history into routine gynecological and urological assessments enables targeted preventive psychological support.

### Imaging biomarkers

Magnetic resonance imaging (MRI) has emerged as a valuable tool for predicting CPPS subtypes. In men with CP/CPPS, periprostatic vein dilatation (detected on pelvic MRI) was found in 63.6% of patients, with 97% specificity and 87% positive predictive value, independent of age, symptoms, and prostatic volume [30]. For endometriosis, specialist endometriosis MRI (eMRI) achieves 91–93.5% sensitivity and 86–87.5% specificity for deep and ovarian endometriosis, making it reliable for surgical mapping and early diagnosis before chronic pain syndromes develop [31, 32]. Transvaginal ultrasound (TVU) also offers a predictive model for pelvic congestion syndrome: a pelvic vein or venous plexus diameter of ≥ 8 mm on TVU has 90% sensitivity and 69% specificity, with an AUC of 0.79 [33].

### Digital biomarkers for health monitoring

Digital biomarkers (DBs); measurable physiological, behavioral, and environmental parameters collected via wearables, smart devices, and medical sensors, are revolutionizing chronic disease management [34]. Continuous monitoring of pain intensity, urinary frequency, sleep patterns, and physical activity enables real-time flare prediction and early intervention. A systematic review of 22 randomized controlled trials involving 2,641 patients with chronic pain conditions (including CPPS, IC/BPS, and fibromyalgia) found that mHealth interventions significantly improved pain intensity, quality of life, and functional disability [35]. Beneficial effects were specifically noted for IC/BPS, CPPS, and endometriosis-associated pain. Remote pelvic yoga programs delivered via videoconference have demonstrated feasibility and acceptability, with average pelvic pain intensity reductions of 1.9 points (worst pain) and 1.1 points (average pain) after two months [36].

## Targeted prevention in primary and secondary care

### Nutraceuticals and mitochondria-relevant compounds

Nutraceuticals are naturally occurring bioactive compounds with health-promoting and illness-preventing properties. Their safety profile and growing demand make them attractive for primary prevention of CPPS, provided they are matched to individual patient profiles [37]. A meta-analysis of 11 randomized controlled trials (975 subjects) found that flavonoids significantly improved the response rate (OR 0.31) and reduced NIH-CPSI scores by 6.96 points in chronic prostatitis patients, with excellent safety profiles [38]. Quercetin, a flavonoid, exerts protective effects in CP/CPPS rat models through inhibition of NF-κB and MAPK signaling pathways, reducing pro-inflammatory cytokines (IL-1β, IL-6, TNFα) and oxidative stress markers [39].

Pollen extracts (e.g., Deprox^®^ 500) have demonstrated significant efficacy in CP/CPPS. A prospective multicenter study of 207 patients showed that pollen extract monotherapy or combination with alpha-blockers produced greater reductions in pelvic pain and urinary symptoms than alpha-blockers alone, while also reducing the need for NSAID rescue medication [40]. Palmitoylethanolamide (PEA), an endogenous fatty acid amide with anti-inflammatory and neuroprotective effects, holds promise for chronic urological pain [41]. Other nutraceuticals with mitochondria-relevant actions include Serenoa repens, pygeum africanum, urtica dioica, lycopene, selenium, astaxanthin, curcumin, and betasitosterol [42].

### Lifestyle and environmental modifications

Patient reports consistently identify cold exposure as a trigger for CP/CPPS flares and symptom aggravation. In a study of 48 men with CP/CPPS, 83% reported that cold caused symptom aggravation or relapse, while 63% obtained relief from hot baths and 46% from spending time in a warm climate [10]. Cold exposure does not need to be prolonged or intense; sitting on cold objects, walking on cold floors, or spending time in cold, damp environments is sufficient [43]. Reflex vasoconstriction in susceptible individuals is a proposed mechanism. Therefore, primary prevention advice for at-risk individuals (e.g. SOP carriers) includes avoiding cold exposure, using perineal heating pads, and seeking warmer climates when possible.

Alcohol intake aggravates CP/CPPS through gut microbiome alterations. A study of 38 CP/CPPS patients found that alcohol consumption led to reduced short-chain fatty acids (propionate and butyrate), increased Th17 cells and IL-17 concentrations, and higher symptom scores [44]. Conversely, patients who quit alcohol showed lower symptom scores and reduced inflammation. Dietary interventions to restore short-chain fatty acid-producing gut flora (e.g., high-fiber diet, resistant starch) represent a preventive strategy.

### Pelvic floor physical therapy and biofeedback

Pelvic floor dysfunction; including urinary and fecal incontinence, pelvic organ prolapse, and chronic pain, can be effectively managed with pelvic floor physical therapy (PFPT) [45]. For CPPS, PFPT focuses on relaxing hypertonic pelvic floor muscles, improving coordination, and reducing trigger points. The National Institute for Health and Care Excellence (NICE) recommends identification of women at high risk for pelvic floor dysfunction using prediction tools, so that preventive advice can be provided early [46]. Capacitive resistive monopolar radiofrequency (CRMRF) at 448 kHz, as an adjunct to physiotherapy, reduced pain scores by more than 2 points and improved quality of life by 5 points in a triple-blind randomized controlled trial of 81 CPPS patients [47].

## Personalized treatment algorithms

### Pharmacological targeting of TRP channels

Transient receptor potential (TRP) channels, particularly TRPV1 and TRPM8, are key mediators of bladder and pelvic pain. In a guinea pig model of non-ulcerative interstitial cystitis, high-threshold muscular afferents were sensitized via TRPV1 up-regulation, high-threshold muscular-mucosal afferents via TRPM8, and mucosal afferents by both [48]. Combination of low-dose TRPV1 and TRPM8 antagonists normalized visceromotor responses to noxious bladder distension, demonstrating therapeutic promise. Although clinical trials are still needed, targeting TRP channels offers a mechanism-based personalized approach for IC/BPS patients with documented TRP channel up-regulation.

### Interventional pain management

For refractory CPPS, interventional strategies include sympathetic and peripheral nerve blocks, chemical and radiofrequency denervation, spinal cord stimulation, and dorsal root ganglion stimulation [49]. A biopsychosocial model should guide these interventions; as psychosocial variables significantly influence outcomes. Gonadal vein embolization for pelvic congestion syndrome provides substantial pain relief in approximately 75% of women, with low repeat intervention rates and minimal complications (coil migration < 2%) [50]. However, evidence quality is low, and patient selection is critical. Percutaneous nephrostomy or double-J stents may be necessary for obstructive anuria secondary to advanced pelvic gynecological cancers, highlighting the importance of early diagnosis to prevent such emergencies [51].

### Traditional Chinese medicine and integrative approaches

Traditional Chinese Medicine (TCM) offers multiple treatment modalities for CP/CPPS, including acupuncture, herbal decoctions, Tuina massage, and external applications. Bibliometric analysis of 106 publications (2003–2025) identified acupuncture as a core intervention, with mechanisms involving immunomodulation, enhanced local blood circulation, reduced inflammatory response, and regulation of the neuroendocrine system [52]. Jiedu Huoxue decoction (JDHXD) improved CP/CPPS recovery in a dose-dependent manner in animal models by activating the Wnt/GSK-3β/β-catenin signaling pathway and inhibiting apoptosis [53]. Electroacupuncture at Guanyuan (CV4), Zhongji (CV3), Huiyang (BL35), and Sanyinjiao (SP6) down-regulated the P2 × 7R/NLRP3 inflammasome pathway, reducing TNF-α, IL1β, and PGE2 in CPPS rats [54]. Qian-Yu decoction (QYD) and its three extracts (polysaccharides, flavonoids, saponins) exerted cooperative anti-inflammatory effects, reducing prostatic index by 44.3% and TNFα by 42.8% [55].

### Emerging and investigational therapies

Low-intensity shock wave therapy (LISWT) has been investigated for CP/CPPS. Although five randomized controlled trials showed possible pain relief, the effect is not consistently maintained over time, and LISWT is not recommended as primary treatment but may be considered as an option for pain relief [56]. Nanoceria (cerium oxide nanoparticles) pretreatment prevented the development of urological chronic pelvic pain syndrome in a cyclophosphamide-induced cystitis mouse model by scavenging reactive oxygen species and down-regulating SerpinB2, the most up-regulated marker in mouse and human UCPPS [57]. T2 peptide, a major autoantigen epitope in experimental autoimmune prostatitis, represents a potential target for immune-based prevention: oral administration of T2-containing soybean trypsin inhibitor prevented CP/CPPS in mice [58, 59].

**Fig. 2:**
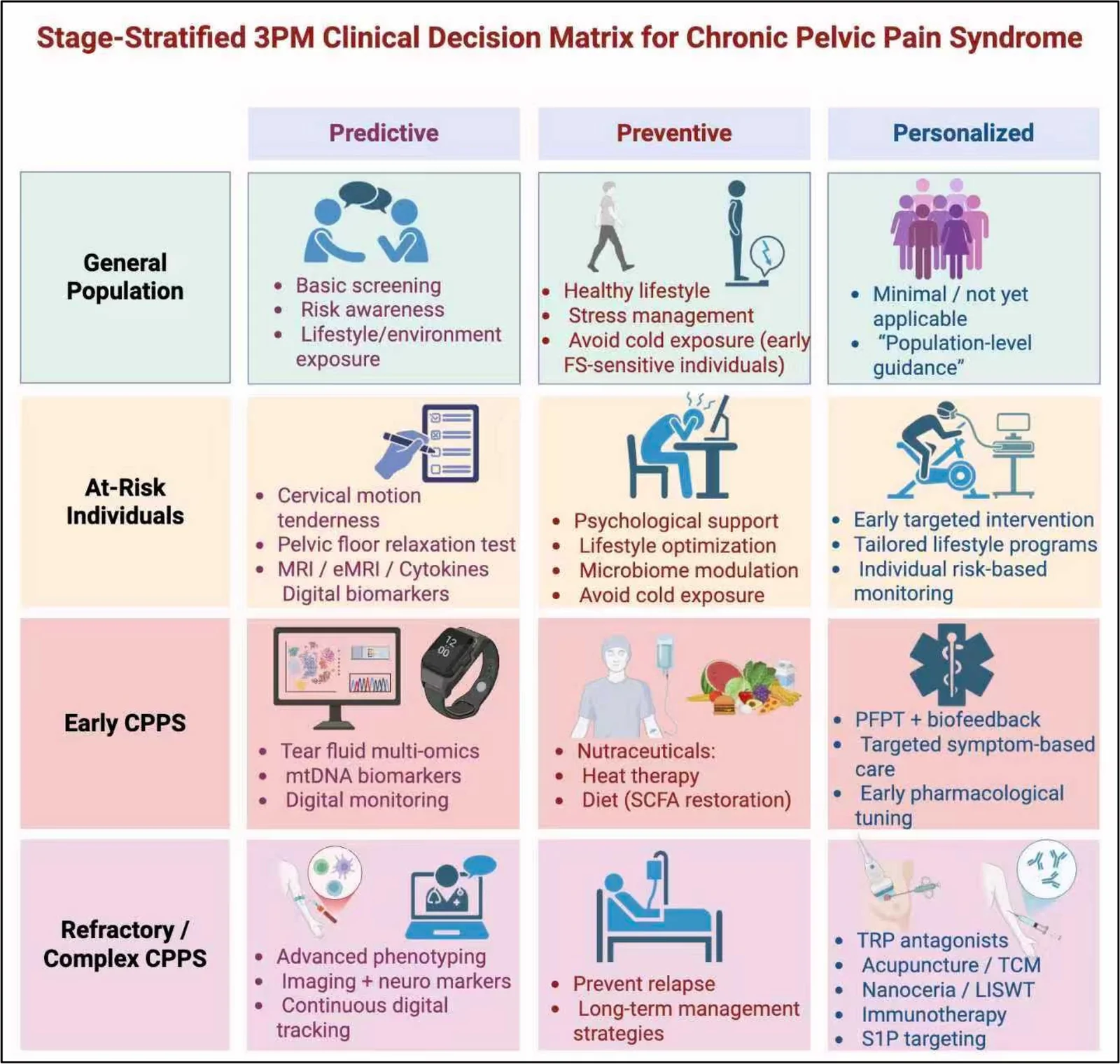
Stage-stratified predictive, preventive, and personalized medicine clinical decision matrix for CPPS. The model integrates four disease stages general population, at-risk individuals, early disease, and refractory/complex CPPS with corresponding predictive diagnostics, targeted prevention, and personalized interventions. Early risk stratification using clinical, imaging, molecular, and digital biomarkers enables timely preventive strategies, including lifestyle optimization and psychological support. In early disease, molecular and digital monitoring guide targeted interventions such as nutraceuticals and pelvic floor therapy. In advanced disease, mechanism-based personalized treatments including neuromodulation, TRP channel targeting, integrative approaches, and emerging therapies are applied. The framework emphasizes continuous monitoring and re-stratification to support adaptive, individualized care across the health-to-disease continuum

Bacillus Calmette-Guérin (BCG) vaccination has been proposed as a therapeutic for endometriosis, based on the hypothesis that peritoneal immune suppression enables implantation of ectopic endometrial tissue, which may be overcome by BCGinduced immune activation [60]. The sphingosine 1-phosphate (S1P) signaling axis mediates neuropeptide S-induced invasive phenotype of endometriotic cells, and targeting SK1/SK2 or S1P1/S1P3 receptors offers non-hormonal therapeutic opportunities [61]. Figure 2 showed stage-stratified predictive, preventive, and personalized medicine clinical decision matrix for CPPS.

### Hormonal modulation: local, systemic and combination strategies in stratified CPPS cohorts

Endocrine modulation is the most extensively trialled pharmacological axis in the estrogen-dependent CPPS subtypes and cannot be omitted from a treatment algorithm claiming comprehensiveness. For endometriosis-associated pain, hormonal suppression is recommended as first-line medical therapy, with agent choice following individual priorities, reproductive intention, comorbidity and tolerability rather than a fixed hierarchy [62]. The evidence is graded rather than uniform: combined oral contraceptives are widely prescribed, yet the supporting randomised evidence is limited in quantity and quality [63], whereas a 2025 Cochrane review of 33 randomised trials (5,059 participants) found that oral progestagens probably reduce overall pain versus placebo (mean difference − 2.58 on a visual analogue scale, 95% CI − 3.13 to − 2.03; moderate certainty) and improve quality of life (SF-36 mean difference 4.11, 95% CI 2.41 to 5.82; high certainty) [64]. Gonadotrophin-releasing hormone (GnRH) agonists relieve pain but impose a hypoestrogenic state that limits treatment duration [65]. The 24-week comparison of dienogest 2 mg/day against depot leuprolide acetate illustrates the trade-off precisely: absolute visual analogue reductions were equivalent (47.5 versus 46.0 mm), while mean lumbar bone mineral density changed by + 0.25% with dienogest against − 4.04% with leuprolide (*p* = 0.0003) [66]. Oral GnRH antagonists have narrowed this gap through dose-titrated, partial estrogen suppression combined with hormonal add-back: elagolix showed dose-dependent responder benefit for dysmenorrhoea and non-menstrual pelvic pain across the replicate Elaris EM-I and EM-II phase 3 trials (872 and 817 women randomised) [67]; relugolix 40 mg with estradiol 1 mg and norethisterone acetate 0.5 mg met both co-primary responder endpoints at 24 weeks in the replicate SPIRIT 1 and SPIRIT 2 trials [68]; and linzagolix 200 mg with add-back reduced both pain domains at three months, whereas 75 mg without add-back improved dysmenorrhoea only [69]. Add-back is therefore an integral component rather than an optional adjunct, and 48-month safety data are now available [70]. Comparable principles govern adenomyosis, managed with progestins, the levonorgestrel-releasing intrauterine system and GnRH analogues [71], while aromatase inhibitors retain a defined niche in refractory or postmenopausal deep disease [72].

Hormonal therapy also carries an explicitly preventive, and hence 3PM-relevant, indication in the peri-operative window. In a systematic review and meta-analysis of 17 studies (2,137 patients), suppression initiated within six weeks of conservative endometriosis surgery reduced recurrence at a median of 18 months by approximately 60% relative to expectant management or placebo (relative risk 0.41, 95% CI 0.26 to 0.65) and lowered subsequent pain scores (standardised mean difference − 0.49, 95% CI − 0.91 to − 0.07) [73]; the post-operative levonorgestrel-releasing intrauterine system has been appraised separately in a dedicated Cochrane review [74], whereas pre-operative treatment carries far weaker support [75]. This is secondary prevention in the strict sense used throughout the present article — an intervention delivered at a defined window of reversibility in a stratified cohort. Its combination with the non-hormonal modalities discussed above is supported in principle by meta-analytic evidence that multidisciplinary care of female chronic pelvic pain outperforms single-discipline treatment for pain (mean difference − 2.19, 95% CI − 3.17 to − 1.22) and sexual function (mean difference 2.47, 95% CI 1.06 to 3.88), though not for quality of life [76]. Decisively for the stratification logic advanced here, hormonal therapy failure is foreseeable: among 114 hormone-naïve patients with endometriosis, higher baseline Central Sensitization Inventory scores and the presence of central sensitisation (CSI ≥ 40; 40.4% of the cohort) were significantly associated with failure to achieve a 30% pain reduction at six months for chronic pelvic pain and deep dyspareunia [77]. Central sensitisation phenotyping, already advocated in this review as a predictive tool, thus doubles as a pre-treatment discriminator between endocrine responders and patients in whom a centrally directed multimodal strategy should be prioritised from the outset.

Local hormonal therapy addresses a distinct and, in the present context, particularly instructive pathway. Genitourinary syndrome of menopause — vulvo-vaginal dryness, burning, dyspareunia and lower urinary tract symptoms arising from mucosal hypoestrogenism — overlaps substantially with the vulvar and bladder pain phenotypes considered above, and is managed predominantly with low-dose vaginal preparations achieving mucosal effect at minimal systemic exposure [78], with comparable efficacy across creams, tablets, pessaries and the estradiol-releasing ring [79]. Non-estrogenic local options extend the range where estrogen is contraindicated or declined: pooled data from three randomised placebo-controlled trials of daily intravaginal prasterone (dehydroepiandrosterone) 6.5 mg in 436 treated versus 260 placebo-treated women showed a 49% reduction in dyspareunia severity alongside objective maturation-index and vaginal-pH improvement (*p* < 0.0001) [80], and oral ospemifene has 12-month efficacy and safety data in vulvar and vaginal atrophy [81]. In provoked vestibulodynia, topical estradiol–testosterone compounding is an established option within expert consensus management [82, 83], whereas combined hormonal contraceptive use has itself been implicated as a modifiable contributor to vulvar pain in susceptible women — an iatrogenic risk that predictive counselling can pre-empt [84]; systemic testosterone in women, conversely, has an evidence-based indication confined to hypoactive sexual desire dysfunction and should not be extrapolated to pelvic pain [85]. The bladder is not exempt from this axis: in 34 premenopausal women with IC/BPS, of whom 94.1% also had vulvodynia, 12 weeks of vulvo-vaginal estriol 0.5 mg cream three times weekly improved urinary and sexual function scores and the vaginal health and maturation indices (*p* < 0.001) [86]. That study is small and uncontrolled, and is therefore hypothesis-generating rather than practice-defining, but it is mechanistically coherent with the documented cyclical variation of bladder pain sensation across the menstrual cycle in interstitial cystitis [87].

The essential role of phenotyping for the most effective targeted treatments can be exemplified by the Flammer syndrome (FS) carriers, in whom vulvar-vaginal dryness is recognized to be phenotypically specific in both pre- and postmenopausal females and manifested despite their usually high estrogen levels. Contextually, estrogen-based treatments are ineffective and could be even harmful for this patient cohort. On the other hand, due to the phenotype-characteristic psychosomatic patterns (SOP), significant stress overload and anxiety negatively impacts both libido and vaginal lubrication in FS carriers [16]. Consequently, the phenotype-targeted approaches considering stress modulation, mitochondrial-based holistic therapies and local lubrication are strongly recommended for the female FS carriers [13, 16, 19, 88].

In the male CPPS patients, the impact of the hormonal axis is considered relatively minor, in general. The 2025 American Urological Association guideline on male chronic pelvic pain does not place endocrine agents among the primary treatments for chronic prostatitis/CPPS [89]. A randomised placebo-controlled trial of finasteride in 76 men with category IIIA CPPS found more men reporting at least mild improvement on active drug (75% versus 54%), yet the authors explicitly declined to recommend finasteride as monotherapy outside coexisting benign prostatic hyperplasia [90]. Androgen signaling in prostatic inflammation remains an active mechanistic field [91], but androgen-directed strategies are at present mechanistically interesting and clinically unproven for pain outcomes. Hormonal therapy in CPPS therefore exemplifies rather than contradicts the key message of this article. It is effective in defined, biologically characterised subgroups and inert or harmful in others; its principal toxicities — bone mineral density loss, vasomotor symptoms, mood change, iatrogenic vulvar pain — are themselves predictable and modifiable through add-back regimens and route selection; and its failure is now measurably foreseeable through central sensitisation phenotyping. Sequential empirical trials of contraceptives, progestins and GnRH analogues until one appears to work is precisely the reactive pattern that the 3PM framework is intended to replace. We therefore recommend that hormonal status and hormone-responsiveness be incorporated explicitly into the stratification matrix presented in the Fig. 2 reproductive stage and estrogen milieu as predictive variables, mucosal hypoestrogenism as a modifiable local risk factor, central sensitisation score as a treatment-response discriminator, and add-back therapy or route modification as individualised risk-mitigation measures. Decisions on specific agents, doses and durations remain the responsibility of the treating clinician in the context of each patient’s fertility intentions, bone health, and thrombotic and oncological risk profile.

## Special populations and comorbidities

### Endometriosis and the “evil twins” syndrome

Endometriosis affects approximately 10% of reproductive-age women and is the most common cause of chronic pelvic pain. However, over 80% of patients with CPP have both endometriosis and interstitial cystitis, a condition termed the “evil twins” syndrome [92]. Failure to look for both conditions leads to delayed diagnosis and unsuccessful therapies. Pelvic MRI and cystoscopy should be considered in endometriosis patients whose pain persists after hormonal or surgical treatment. Endometriosis also co-occurs with polycystic ovary syndrome (PCOS) in 1 in 20 women undergoing laparoscopy, and these patients have a ten-fold higher risk of subfertility [93]. Long COVID is significantly associated with endometriosis (pooled RR 1.41), suggesting that chronic inflammatory states predispose to postviral complications [94].

### Hypermobile Ehlers-Danlos syndrome and hypermobility spectrum disorders

Hypermobile Ehlers-Danlos syndrome (hEDS) and hypermobility spectrum disorders (HSD) are the most common symptomatic joint hypermobility conditions seen in clinical practice [95]. They are frequently associated with chronic pain, fatigue, orthostatic intolerance, functional gastrointestinal disorders, and pelvic and bladder dysfunction. Management requires a multidisciplinary team including physical therapy, psychological support, and self-management education. Orthopedic surgery for joint instability is controversial due to complications; non-operative treatment should be exhausted first [96].

### Post-vasectomy pain syndrome

Post-vasectomy pain syndrome (PVPS) affects a subset of men and is often underrecognized. Etiology is multifactorial (immunological, mechanical, neuropathic). Initial management is conservative (NSAIDs, neuropathic agents, pelvic floor therapy). For refractory cases, microsurgical spermatic cord denervation, vasectomy reversal, or epididymectomy may be considered [97]. Prevention through informed consent and meticulous surgical technique is key.

### Ureterolithiasis

Ureterolithiasis presents with colicky flank pain radiating to the groin, often with hematuria (85%). While most stones ≤ 5 mm pass spontaneously, those > 7 mm or not moving in 4–6 weeks may require ureteroscopy with laser lithotripsy or extracorporeal shock wave lithotripsy [98]. Obstructive pyelonephritis with infection requires urgent surgical drainage. Long-term prevention with 24-hour urine collection is recommended for high-risk patients (recurrent stone formers, solitary kidney, cystine stones, immunocompromised). Notably, ureterolithiasis is associated with cardiovascular disease, diabetes, metabolic syndrome, and obesity, conditions also linked to mitochondrial stress and sympathetic overdrive [99].

## Flares: prediction, prevention, and management

Flares; acute exacerbations of pelvic pain and urinary symptoms, are a hallmark of CPPS. In the MAPP Research Network longitudinal study of 385 participants, 75.8% reported at least one flare over 11 months, with a median incidence rate of 0.13 per biweekly assessment [100]. Flare duration varied from 1 to 150 days, with 94.3% of variability within individuals. Women, patients with worse non-flare symptoms, bladder hypersensitivity, and chronic overlapping pain conditions experienced more frequent, severe, and longer-lasting flares.

Triggers for flares are individual- or group-specific rather than universal. In a case-crossover study of 292 participants, only recent sexual activity (OR 1.44) and urinary tract infection symptoms (OR 3.39) were associated with flare onset in the full sample [101]. However, subgroup analyses revealed associations with certain dietary factors, abdominal muscle exercises, and vaginal infection-like symptoms. Rising pollen count (above medium thresholds) triggered flares in participants with allergies or respiratory disorders (OR 1.31) [102]. These findings caution against rigid, global flare prevention strategies and support individualized trigger identification.

Flare frequency is independently associated with illness impact and healthcare-seeking activity, even after adjusting for typical non-flare pain levels [103]. Patients who experience one or more flares per day have significantly worse general illness impact. Qualitative research with male UCPPS patients identified themes of social isolation, decreased sexual activity, reduced travel, and potential loss of employment due to flares and the threat of flares [104]. Therefore, reducing flare frequency; even without changing mean pain intensity, should be a treatment goal.

Management strategies for flares include analgesics, thermal therapy (heat application), rest, and in some cases, rescue medications. However, few strategies have empirical support. Prophylactic approaches include trigger avoidance (cold, alcohol, certain foods), pelvic floor relaxation techniques, and stress reduction.

## The microbiome as a therapeutic target in stratified CPPS patients

**Fig. 3:**
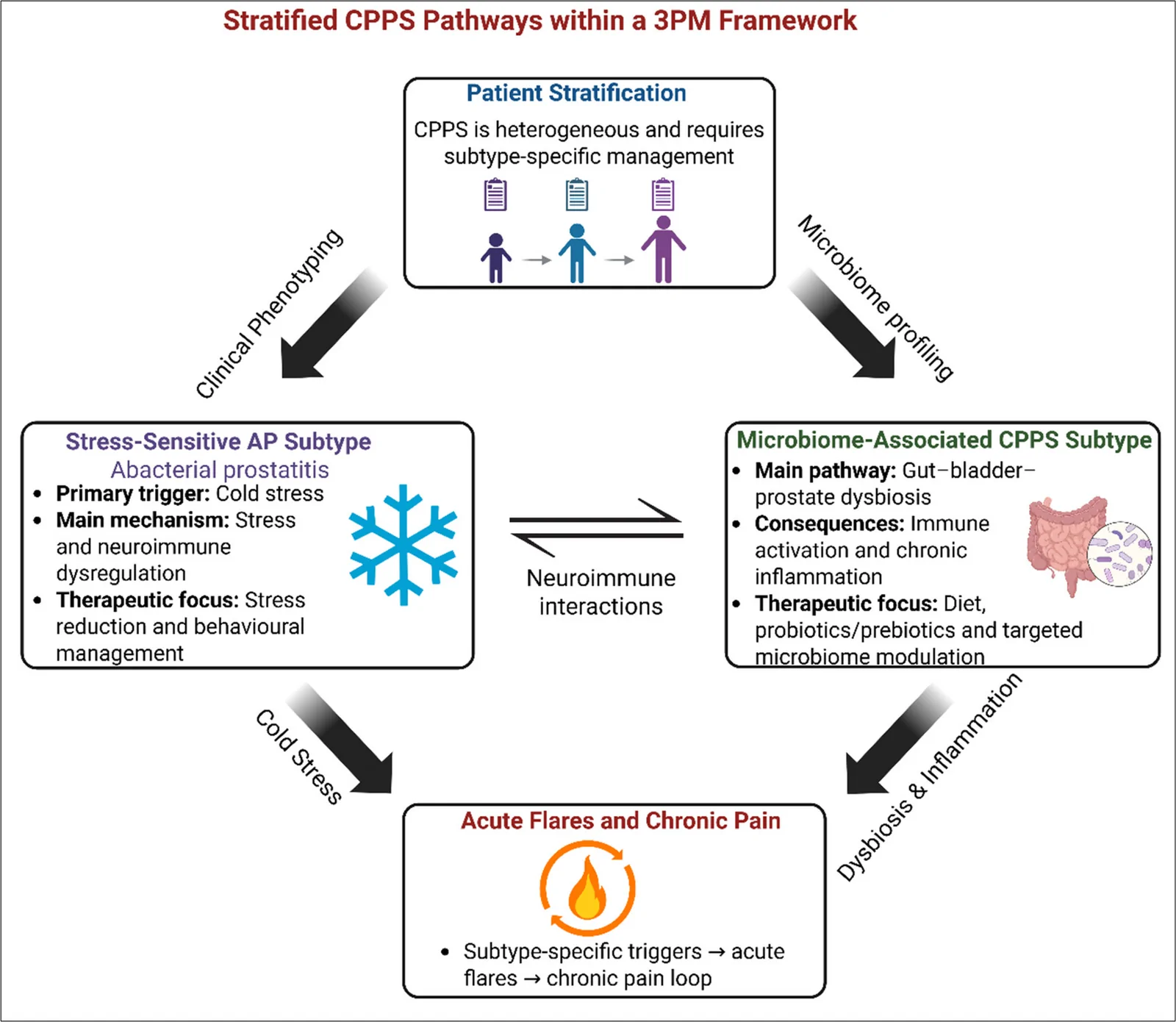
Stratified CPPS pathways within a 3PM framework. Conceptual model illustrating how patient stratification enables personalized management of chronic prostatitis/chronic pelvic pain syndrome (CPPS). Clinical phenotyping and microbiome profiling identify two major endotypes: a stress-sensitive abacterial prostatitis (AP) subtype, in which cold stress and neuroimmune dysregulation predominate and stress-focused interventions are prioritized, and a microbiome-associated subtype characterized by gut–bladder–prostate dysbiosis, immune activation, and microbiome-targeted therapies. Bidirectional neuroimmune interactions link both pathways, which ultimately converge on acute flares and chronic pain, supporting a Predictive, Preventive, and Personalized Medicine (3PM) approach to CPPS management

Considering a role of microbiome in the CPPS risks, it is essential to emphasize that patient stratification is crucial for the targeted CPPS treatments within this extremely heterogeneous patient subpopulation. For example, for the CPPS patients suffering specifically from the abacterial prostatitis (AP) and characterized by pain, voiding and sexual dysfunction persisting for over 3 months, the cold stress provocation appears to be one of the most specific disease triggers resulting in the CPPS manifestation [10]. At the diagnostics and treatment levels, this stratified patient cohort has to be differentiated from the below described microbiome-relevant patient cohorts. Consequently, any application of antibiotics and/or microbiome modulators are not considered the primary therapeutic target for the AP-patients in contrast to their increased stress sensitivity which appears to be the most effective target relevant for the SOP subpopulation in general as discussed above. Figure 3 illustrates a 3PM framework for CPPS, highlighting how patient stratification guides personalized management through distinct stress-sensitive and microbiome-associated disease pathways.

The human body hosts trillions of microorganisms that collectively form the microbiome, a dynamic ecosystem increasingly recognized as a key modulator of health and disease. For decades, the urinary tract was considered sterile, but advanced sequencing technologies have overturned this dogma. Today, it is established that the healthy urinary bladder harbors a low-biomass but functionally relevant microbial community, and alterations in this community; termed dysbiosis, are associated with several urological and gynecological conditions, including chronic pelvic pain syndrome [105, 106]. Understanding the microbiome’s role in CPPS opens new avenues for predictive diagnostics, targeted prevention, and personalized treatment within the 3PM framework.

In interstitial cystitis/bladder pain syndrome (IC/BPS), early studies using catheterized urine suggested differences in microbial composition between patients and controls, but results were inconsistent due to contamination risks and low bacterial loads. A landmark study by Wolfe and colleagues addressed this limitation by analyzing bladder biopsy tissues from 30 women with IC/BPS and 10 controls using 16 S rRNA gene sequencing and fluorescence in situ hybridization [106]. Bacteria were detected in most biopsies, with *Staphylococcus* being the most abundant genus, followed by *Lactobacillus* and *Escherichia*. However, there was no significant difference in overall microbial composition between IC/BPS patients and controls. Importantly, bacteria were visualized within urothelial cells, shed cells in the urothelium, and capillary walls in the lamina propria, indicating that the bladder mucosa harbors a resident microbiota that is not simply a contaminant from the urethra or vagina. These findings suggest that the presence of bacteria alone does not explain IC/BPS; rather, the functional state of the microbiome (e.g., metabolic activity, virulence factors, host immune interaction) may be more relevant than taxonomic composition.

For some stratified chronic prostatitis/chronic pelvic pain syndrome (CP/CPPS), the gut–prostate axis, indeed, may represent a critical pathway. A study by Liu and colleagues demonstrated that alcohol intake aggravates CP/CPPS through gut microbiome alterations [44]. Specifically, alcohol consumption reduced the abundance of short-chain fatty acid (SCFA)-producing bacteria, leading to decreased propionate and butyrate levels. These SCFAs normally maintain intestinal barrier integrity and exert anti-inflammatory effects via G-protein coupled receptors. Their reduction resulted in increased Th17 cell differentiation, elevated interleukin-17 concentrations, and higher symptom scores on the NIH-CPSI. Notably, patients who quit alcohol showed partial restoration of SCFA levels and clinical improvement. This mechanistic link provides a strong rationale for dietary and microbiome-targeted interventions in CP/CPPS prevention and management.

Beyond the gut, the intraprostatic microbiome itself may influence disease. Studies have identified bacterial DNA within prostatic tissue from men with CP/CPPS, although no single pathogen is consistently found. The concept of “dysbiosis” rather than infection is gaining traction: an imbalance between commensal and potentially pathogenic organisms may trigger low-grade chronic inflammation that sensitizes pelvic nerves and promotes pain [107–109]. Similarly, in benign prostatic hyperplasia (BPH) and prostate cancer, alterations in the gut and urinary microbiomes have been linked to disease progression and treatment response, suggesting shared pathways across prostatic disorders [110].

The therapeutic implications of these findings are substantial. First, restoration of SCFA-producing gut flora through dietary fiber (e.g., resistant starch, inulin, pectin) represents a low-risk, cost-effective preventive strategy for individuals at risk of CP/CPPS, particularly those with alcohol use or high-fat, low-fiber diets. Second, probiotic supplementation with specific strains that produce butyrate (e.g., *Faecalibacterium prausnitzii*, *Eubacterium rectale*) or that modulate Th17 responses is an area of active research, though clinical trials in CPPS are lacking. Third, prebiotics (e.g., fructooligosaccharides, and galactooligosaccharides) can selectively stimulate beneficial bacteria. Fourth, fecal microbiota transplantation (FMT) has shown promise in other chronic inflammatory conditions (e.g., ulcerative colitis, irritable bowel syndrome) and could be investigated for refractory CP/CPPS, especially in patients with documented dysbiosis and failed conventional therapies [111].

Importantly, the urinary microbiome itself may be modifiable. Cranberry (*Vaccinium macrocarpon*) extracts, long used for urinary tract infection prevention, alter urothelial adhesion of bacteria and have anti-inflammatory properties. While direct evidence in IC/BPS is limited, cranberry’s effects on the urinary microbiome warrant investigation [105]. Similarly, D-mannose, a simple sugar that inhibits bacterial adherence, has been studied in recurrent cystitis but not specifically in CPPS. Given the overlap between IC/BPS and chronic urinary tract infections, personalized assessment of the urinary microbiome using next-generation sequencing could identify patients who might benefit from targeted antimicrobial or probiotic therapy, moving beyond empirical antibiotic trials that often fail and contribute to resistance.

A critical gap remains the lack of standardized methods for urinary microbiome sampling. Voided urine is contaminated by vaginal and periurethral flora; catheterized urine reduces but does not eliminate contamination; and biopsy samples are invasive. Despite these challenges, the field is moving toward consensus protocols. The EPMA position on digital biomarkers highlights the potential of integrating microbiome data with other omics (metabolomics, proteomics) and wearable sensor data to create comprehensive individual patient profiles [34]. Machine learning algorithms can identify microbial signatures that predict flare risk or treatment response, enabling truly personalized microbiome-based interventions.

In summary, the microbiome is not a peripheral curiosity but a central player in CPPS pathogenesis. The gut–bladder–prostate axis operates through SCFAs, immune modulation, and neural pathways. Preventive strategies targeting the microbiome; dietary modification, alcohol cessation, probiotics, prebiotics, and potentially FMT, should be incorporated into 3PM-guided care. Future research must prioritize longitudinal studies that track microbiome changes before, during, and after CPPS onset, as well as randomized controlled trials of microbiome-based therapies.

**Fig. 4:**
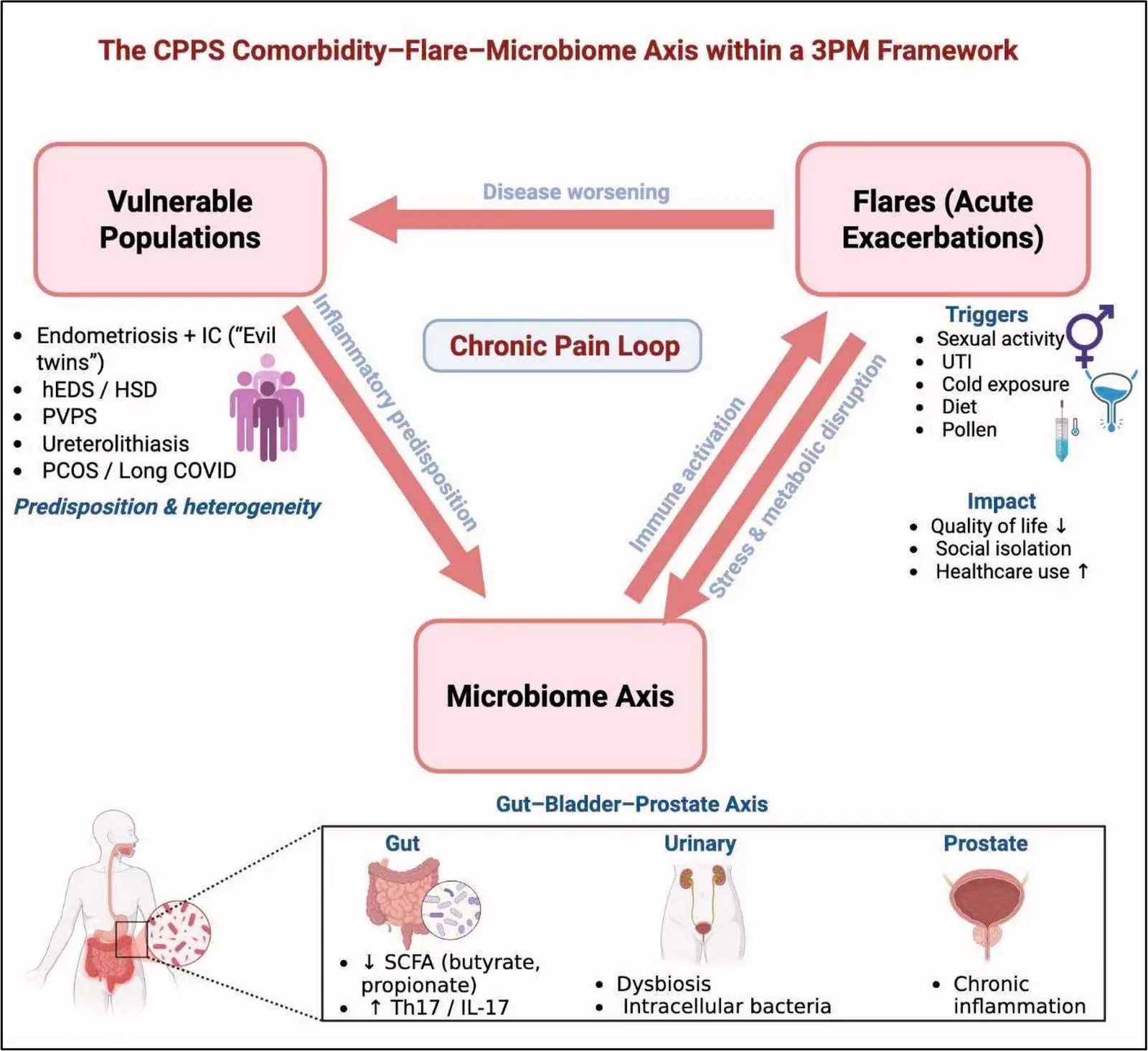
The comorbidity–flare–microbiome axis in chronic pelvic pain syndrome (CPPS) within a 3PM framework. Vulnerable populations interact bidirectionally with flares and microbiome alterations across the gut–bladder–prostate axis. Dysbiosis, reduced short-chain fatty acids, and immune activation drive symptom exacerbation, while flares and comorbidities reinforce disease progression through a chronic pain loop. This framework supports prediction of flare risk and personalized microbiome-informed interventions

Figure 4 summarizes the comorbidity–flare–microbiome axis in chronic pelvic pain syndrome (CPPS) in the 3PM context.

## Implementation of 3PM in clinical practice: barriers and solutions

Despite the compelling scientific rationale for predictive, preventive, and personalized medicine in chronic pelvic pain syndrome, translation from research to routine clinical practice faces substantial barriers. These obstacles are not unique to CPPS but are amplified by the syndrome’s heterogeneous presentation, historical neglect, and the fragmented nature of healthcare delivery across urology, gynecology, pain medicine, physiotherapy, and psychology. Understanding these barriers is the first step toward designing effective implementation strategies.

A qualitative study by Li and colleagues interviewed 12 health practitioners from multiple disciplines who manage male CP/CPPS patients [112]. Through reflexive thematic analysis, they identified five broad, interrelated themes. The first theme, “Where to Start,” reflects diagnostic uncertainty. Unlike conditions with clear biomarkers (e.g., prostate-specific antigen for prostate cancer), CPPS lacks a gold standard test. Practitioners reported feeling overwhelmed by the multitude of possible contributing factors; infection, inflammation, pelvic floor dysfunction, central sensitization, psychological distress, and unsure which to address first. This diagnostic paralysis often leads to a trial-and-error approach that frustrates both clinicians and patients.

The second theme, “Insufficient Resources,” highlights the scarcity of specialized services. Pelvic floor physical therapy (PFPT) is evidence-based for CPPS, yet many regions have few trained pelvic health physiotherapists, and insurance coverage is inconsistent. Similarly, access to pain psychologists, multidisciplinary pain clinics, and advanced imaging (e.g., pelvic MRI with endometriosis protocols) is limited. Even when resources exist, long waiting times discourage timely intervention. The third theme, “Prioritization,” reflects that CPPS is often deprioritized in healthcare systems relative to acute conditions (e.g., trauma, infection) or life-threatening diseases (e.g., cancer). Patients with chronic pelvic pain may wait months for a specialist appointment, and consultations are often rushed, leaving little time for the biopsychosocial assessment that 3PM requires.

The fourth theme, “Training and Confident Practice,” is particularly important. Many urologists, gynecologists, and primary care physicians received little to no formal training in chronic pain management, central sensitization, or the biopsychosocial model. They may feel confident managing acute bacterial prostatitis or a simple ovarian cyst but lack skills in motivational interviewing, cognitive-behavioral techniques, or interpreting digital biomarker data. Consequently, they default to prescribing analgesics or antibiotics, even when evidence for their efficacy in CPPS is weak. The fifth theme, “Constraints in Help-Seeking,” pertains to patients: shame, stigma, and difficulty articulating pelvic symptoms delay presentation, and when patients do seek help, they may encounter disbelief or dismissal, particularly if they have a history of trauma.   

Beyond these qualitative findings, additional barriers include the high cost and limited reimbursement for 3PM tools. Tear fluid multi-omics, AI-based data interpretation, and mitochondrial biosensorics are not yet covered by most insurance plans, making them accessible only in research settings or to wealthy patients. Digital biomarkers require patients to own and use wearables or smartphones, creating a digital divide. Furthermore, the regulatory pathway for approving AI-based diagnostic algorithms is still evolving, and many clinicians are skeptical of “black box” recommendations.

Despite these challenges, solutions are emerging. The EPMA’s roadmap for digital biomarker implementation provides a template [34]. Key strategies include: (1) Education and training – integrating 3PM principles and chronic pain management into medical school curricula and continuing professional development, with hands-on workshops on pelvic examination techniques (e.g., parametropathy testing, pelvic floor muscle assessment). (2) Clinical decision support tools – embedding validated prediction models (e.g., body map for widespread pain, flare frequency questionnaire, periprostatic vein dilatation criteria) into electronic health records, with automatic prompts for preventive interventions. (3) Task shifting and interdisciplinary collaboration – training nurse practitioners, physiotherapists, and psychologists to deliver specific components of 3PM care (e.g., PFPT, cognitive-behavioral therapy, nutraceutical counseling), with urologists and gynecologists serving as coordinators rather than sole providers. (4) Telehealth and mHealth – remote delivery of pelvic yoga, biofeedback, and cognitivebehavioral therapy has been shown feasible and acceptable, reducing geographical and financial barriers [35, 36]. (5) Value-based payment models – shifting from fee-for-service to bundled payments or capitation that reward prevention and long-term outcomes rather than procedures and consultations. Early evidence suggests that 3PM-guided care for CPPS reduces unnecessary surgeries, emergency department visits, and opioid prescriptions, offsetting the upfront costs of predictive diagnostics.

Another critical implementation lever is patient empowerment. Digital health monitoring allows patients to track their own symptoms, identify flare triggers, and see the impact of lifestyle modifications in real time. This active role enhances self-efficacy and adherence. Patient support groups and online communities can provide peer validation and practical tips, complementing professional care.

Finally, terminology standardization is essential for implementation. The recent joint report from the International Urogynecological Association and the American Urogynecologic Society recommends replacing “interstitial cystitis” with “female bladder pain syndrome” (FBPS) to avoid the misleading implication of interstitial inflammation and to align with the broader understanding of chronic pain as a central phenomenon [12, 16, 113]. Clear, consistent terminology facilitates coding, referral pathways, and patient communication.

In conclusion, implementing 3PM for CPPS is not a distant dream but a practical necessity. The barriers are real but surmountable through targeted education, resource allocation, digital health monitoring, and payment reform. The cost of inaction (continued suffering, disability, opioid misuse, and healthcare waste) far exceeds the investment needed to transform care.

## Conclusions and expert recommendations

Chronic pelvic pain syndrome represents a paradigm case for the urgent transition from reactive symptom suppression to predictive, preventive, and personalized (3PM) medicine. The evidence synthesized in this review establishes that CPPS is not merely a peripheral organ pathology but a systemic disorder driven by sympathetic overdrive, mitochondrial stress, central sensitization, and microbiome alterations, all of which are detectable and modifiable before a clinical manifestation of the disease. The recognition that Flammer Syndrome phenotype carriers and individuals with sympathetic overdrive are predisposed to CPPS opens unprecedented opportunities for early risk stratification and primary risks’ mitigation such as lifestyle optimization, stress modulation, and targeted nutraceutical interventions.

**Fig. 5:**
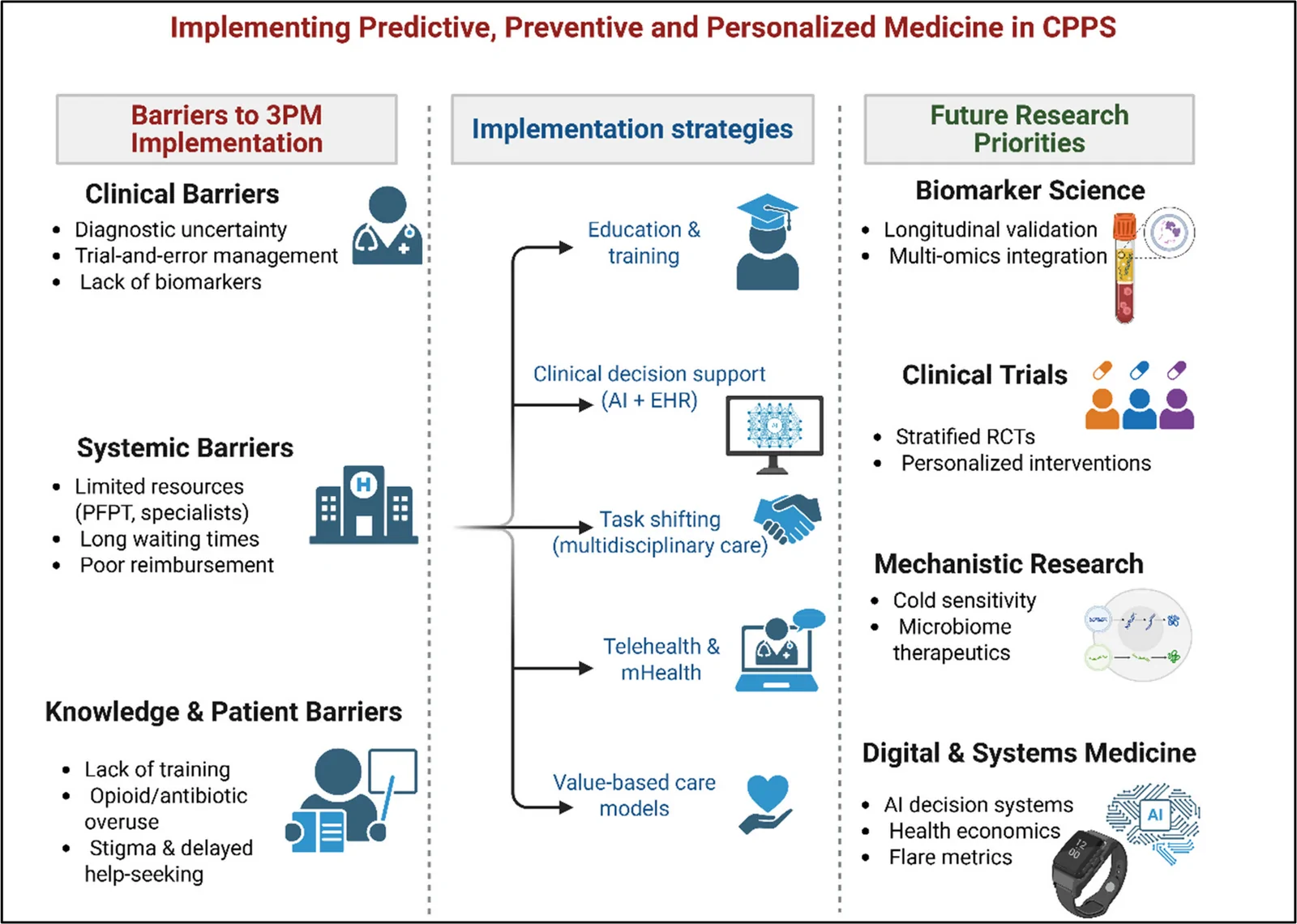
Translational framework for implementing predictive, preventive, and personalized medicine in CPPS. Key barriers including diagnostic uncertainty, limited resources, insufficient training, and patient-related constraints impede effective care. These challenges are addressed through targeted implementation strategies such as education and training, AI-enabled clinical decision support, multidisciplinary care, telehealth integration, and value-based models. The framework is supported by priority research domains, including biomarker validation, stratified clinical trials, mechanistic studies, and digital health innovations, which collectively enable a shift toward personalization, data-driven CPPS management

Implementation of 3PM-guided care summarized in Fig. 5 requires overcoming barriers including advanced population screening, multi-professional expertise, diagnostic uncertainty, and fragmented healthcare delivery, amongst others. However, emerging tools; tear fluid multi-omics, mitochondrial biosensorics, digital health monitoring, AI-based data interpretation, and pelvic MRI, now enable comprehensive individualized patient profiling at the primary care level. Personalized treatment algorithms integrating modulation of mitochondrial homeostasis, TRP channel antagonists, pelvic floor physiotherapy, microbiome restoration, and integrative approaches offer mechanism-based solutions for stratified patient cohorts. The cost of maintaining reactive care, continued suffering, disability, opioid dependence, and healthcare waste, far exceeds the investment needed to transform CPPS management. By embracing the 3PM framework, healthcare systems can protect vulnerable individuals against health-to-disease transition, prevent disease progression, and deliver patient-centered, cause-oriented, and cost-effective proactive care for chronic pelvic pain syndrome.

## Data Availability

No datasets were generated or analysed during the current study.

## Code availability

All protein and gene accession codes are available in the Swiss-Prot and GenBank databases.

## Abbreviations

3PM (PPPM): Predictive, preventive and personalised medicine
AI: Artificial intelligence
AP: Abacterial prostatitis
BCG: Bacillus Calmette‑Guérin
CP/CPPS: Chronic prostatitis / chronic pelvic pain syndrome
CPPS: Chronic pelvic pain syndrome
DBs: Digital biomarkers
eMRI: Endometriosis MRI
ET-1: Endothelin-1
FBPS: Female bladder pain syndrome
hEDS: Hypermobile Ehlers‑Danlos syndrome
HSD: Hypermobility spectrum disorders
IC/BPS: Interstitial cystitis / bladder pain syndrome
LISWT: Low‑intensity shock wave therapy
PAF: Peak alpha frequency
PCOS: Polycystic ovary syndrome
PEA: Palmitoylethanolamide
pPPP: Prolonged post‑operative pelvic pain
PVPS: Post‑vasectomy pain syndrome
S1P: Sphingosine 1‑phosphate
TCM: Traditional Chinese Medicine
TRP: Transient receptor potential (channels)
TVU: Transvaginal ultrasound
